# Utility of needle biopsy in centrally located lung cancer for genome analysis: a retrospective cohort study

**DOI:** 10.1186/s12890-023-02749-1

**Published:** 2023-12-01

**Authors:** Kei Kunimasa, Shingo Matsumoto, Keiichiro Honma, Motohiro Tamiya, Takako Inoue, Takahisa Kawamura, Satoshi Tanada, Akito Miyazaki, Ryu Kanzaki, Tomohiro Maniwa, Jiro Okami, Yuji Matsumoto, Koichi Goto, Kazumi Nishino

**Affiliations:** 1 Department of Thoracic Oncology, Osaka International Cancer Institute, 3-1-69 Otemae Chuoku, Osaka City, Osaka, 541-8567 Japan; 2 Department of Thoracic Oncology, National Cancer Center Hospital East, Kashiwa, Japan; 3 Department of Diagnostic Pathology & Cytology, Osaka International Cancer Institute, Osaka, Japan; 4 Department of Clinical Laboratory, Osaka International Cancer Institute, Osaka, Japan; 5 Department of General Thoracic Surgery, Osaka International Cancer Institute, Osaka, Japan; 6 Department of Endoscopy, Respiratory Endoscopy Division, National Cancer Center Hospital, Tokyo, Japan

**Keywords:** Lung cancer diagnosis, Next-generation sequencing, Sampling method, NGS success rate, Re-biopsy

## Abstract

**Background:**

It is essential to collect a sufficient amount of tumor tissue for successful next-generation sequencing (NGS) analysis. In this study, we investigated the clinical risk factors for avoiding re-biopsy for NGS analysis (re-genome biopsy) in cases where a sufficient amount of tumor tissue could not be collected by bronchoscopy.

**Methods:**

We investigated the association between clinical factors and the risk of re-genome biopsy in patients who underwent transbronchial biopsy (TBB) or endobronchial ultrasound-guided transbronchial needle aspiration (EBUS-TBNA) and required re-genome biopsy in cases enrolled in LC-SCRUM Asia, a prospective nationwide genome screening project in Japan. We also examined whether the frequency of re-genome biopsy decreased between the first and second halves of the enrolment period.

**Results:**

Of the 572 eligible patients, 236 underwent TBB, and 134 underwent EBUS-TBNA. Twenty-four TBBs required re-genome biopsy, and multivariate analysis showed that the risk of re-genome biopsy was significantly increased in lesions where the tumor lesion was centrally located. In these cases, EBUS-TBNA should be utilized even if the lesion is a pulmonary lesion. However, it should be noted that even with EBUS-TBNA, lung field lesions are at a higher risk of re-canalization than mediastinal lymph node lesions. It was also found that even when tumor cells were detected in rapid on-site evaluation, a sufficient amount of tumor tissue was not always collected.

**Conclusions:**

For centrally located pulmonary mass lesions, EBUS-TBNA, rather than TBB, can be used to obtain tumor tissues that can be analyzed by NGS.

## Background

The rapid identification of targetable driver alterations is crucial for treatment decision-making in advanced-stage lung cancer [1–3]. Molecular-targeted drugs often exhibit a good response rate. In cases of advanced-stage lung cancer, where the disease progresses rapidly, it’s imperative to identify alterations promptly [4]. The use of next-generation sequencing (NGS) or multiplex real-time (RT) and reverse-transcription-polymerase chain reaction (PCR) is essential for the identification of the alterations, and the collection of a sufficient amount of tumor tissue is essential for successful tests [5–7]. However, for many advanced-stage lung cancer cases, collecting tumor tissue predominantly depends on bronchoscopy, which is often more challenging than surgical biopsy [8, 9]. Histopathological evaluation, which assesses the tumor content of specimens collected via bronchoscopy, often takes several days [10]. Therefore, the need for re-genome biopsy leads to a significant loss of time.

Bronchoscopy is often a stressful procedure, even when general anesthesia is used [11]. The number of biopsies required to obtain a sufficient amount of tissue is high, which may eliminate the effects of local anesthesia in the pharynx [12, 13]. Coughing during testing is considered to be one of the most significant causes of patient distress [14]. With the use of epidermal growth factor receptor (EGFR)-tyrosine kinase inhibitors (TKIs), the earliest molecular targeted agents introduced in lung cancer, re-biopsy of resistant lesions has become popular with the aim of identifying emerging *EGFR* T790M as a resistance mutation to first- and second-generation EGFR-TKIs [15, 16]. However, a small number of patients refuse to undergo re-biopsy due to the pain of bronchoscopy, and it is clear that repeated bronchoscopy should be avoided, both in terms of patient burden and burden on healthcare providers.

Bronchoscopic biopsies for successful genetic analysis differ from diagnostic biopsies in terms of the quantity of tumor tissue specimens collected. We have made special efforts to perform these biopsies as “genome biopsies” [8, 10]. Before starting sampling for genetic analysis, it is important to consider the method and site from which the biopsy will be taken [17]. Consider the sites where a surgical biopsy can be performed, and if bronchoscopy is used, assess whether the biopsy should be performed from the primary lung field or from mediastinal lymph node metastases. Although these efforts have improved the success rate of genetic analysis, including NGS analysis of bronchoscopic biopsies, there are still some cases that require “re-genome biopsy.” In the present study, we analyzed the risk factors for re-genome biopsy with bronchoscopy for successful genetic analysis of lung cancer cases from our own data.

## Materials and methods

### Patients and clinical characteristics

This study was conducted among patients enrolled in LC-SCRUM (The lung cancer genomic screening project for individualized medicine)-Asia from 04/01/2019 to 30/11/2022 at our institution. LC-SCRUM-Asia (UMIN ID: UMIN000036871), formerly known as LC-SCRUM-Japan (UMIN ID: UMIN000010234), is a prospective, nationwide clinical and genomic screening program for lung cancer and the inclusion criteria was previously described [18–20]. The date of first registration in LC-SCRUM- Japan is 13/03/2013 and in LC-SCRUM Asia is 01/06/2019. All patients provided written informed consent for enrolment in the LC-SCRUM-Asia program. LC-SCRUM Asia only covers non-small cell lung carcinoma and not other cancer types. This project used NGS-based genetic analysis primarily for fresh-frozen specimens. The flow of genetic analyses in LC-SCRUM Asia has been reported previously. The registration period began in January 2019 because the specimen collection methods for genetic alteration analysis at our institution were standardized from the same month [8].

Data on the age, sex, histopathological diagnosis, stage based on imaging studies, and genetic alteration analysis of the enrolled patients in LC-SCRUM Asia were retrospectively extracted from their electronic medical records. Based on the location, the primary lung tumor was categorized as central or peripheral using chest computed tomography (CT) imaging before treatment initiation. The criteria for categorization as central and peripheral were as follows: central location was defined as within 2 cm of the proximal bronchial tree based on the Radiation Therapy Oncology Group (RTOG) criteria, or within 2 cm of the heart, trachea, pericardium, or vertebral bodies but 1 cm away from the spinal canal based on a modification of the MD Anderson Cancer Center definition [21, 22]. In addition, tumor localization and the maximum diameter of the primary lesion in the lung were evaluated in the right upper, middle and left lobes and left upper and lower lobes. Imaging evaluations were independently assessed by two researchers and discussed in case of differing views.

### Sampling methods for NGS analysis in LC-SCRUM Asia

In LC-SCRUM Asia, primarily fresh-frozen human specimens obtained by each method were submitted. A total of 100 mL of body fluid specimens were permitted. NGS was performed after the presence of tumor cells was confirmed. Sampling methods for NGS analysis, which we called “genome biopsy,” are being performed at our institution since January 2019 [8]. The details of this process have been previously reported [8, 10]. The overview is as follows: first, surgical biopsy specimens were preferentially submitted in collaboration with pathologists and surgeons; second, during sampling using transbronchial biopsy (TBB), standard biopsy forceps (FB-231D. A; Olympus, Tokyo, Japan) with a 5.0-mm cup opening were used; third, during sampling using endobronchial ultrasound-guided transbronchial needle aspiration (EBUS-TBNA), biopsies were performed using a 22-gauge needle at least twice whenever possible; fourth, for CT-guided biopsy, two samples were submitted; fifth, before and after sampling with transbronchial biopsy for NGS analysis, specimens were also collected for pathological analysis to confirm that the freshly obtained samples contained tumor cells; and finally, all samples were submitted following specimen evaluation by pathologists. For specimens collected through bronchoscopy, if the presence of viable tumor cells could not be confirmed in both samples collected before and after the frozen samples were submitted, or if viable tumor cell content was ≤10%, NGS analysis was not expected to succeed and the specimen was not submitted (tissue confirmation) [10]. In these cases, a second biopsy, which was called “re-genomebiopsy,” was performed by changing the sampling method, as necessary [10]. All bronchoscopists who performed bronchoscopy were certified by the Japan society for respiratory endoscopy.

### Genome biopsy and re-genome biopsy

In lung cancer cases, a genome biopsy is a biopsy to obtain a sufficient amount of tumor tissue specimen not only to confirm the diagnosis but also to perform a successful gene panel analysis [8, 10]. Unlike diagnostic biopsies, genome biopsy requires multiple biopsies, which lengthens the examination time and increases the burden on the patient as the effects of anesthesia wear off [17]. The specific biopsy procedure is as described above. It is important to identify lesions that can tolerate multiple biopsies and to determine the optimal biopsy method, including surgical biopsy [17]. Even if a diagnosis is confirmed, another biopsy may be necessary if sufficient samples have not been collected for gene panel analysis. In this study, biopsies in such cases are referred to as “re-genome biopsy.

### Rapid on-site evaluation (ROSE) of the cytology aspirate

ROSE was performed using the modified Gill-Shorr method [23]. For all specimens collected by TBB and EBUS-TBNA, ROSE was performed on the cytology sample obtained from the first biopsy specimen, and the specimen was collected for genetic analysis if tumor cells were confirmed. Until tumor cells were confirmed, the biopsy was continued or switched to another biopsy method.

### DNA and RNA extraction and definition of analysis success

DNA/RNA was extracted and purified using a nucleic acid extraction kit (AllPrep DNA/RNA Mini Kit; QIAGEN) according to the manufacturer’s protocol as previously described [8, 10]. The DNA/RNA concentrations were quantified using the Qubit fluorometric assay (Thermo Fisher Scientific). The target region was amplified using multiplex PCR for DNA and RNA, and somatic mutations in the region were detected. Hotspot mutations (single-nucleotide variants, deletions, and insertions) and copy number variations were detected in the DNA-based sequences, and fusion gene alterations were detected in the RNA-based sequences. In this study, analysis failure was considered when NGS analysis was not accomplished owing to insufficient DNA or RNA sample volumes.

### NGS analysis in LC-SCRUM Asia

The Oncomine Comprehensive Assay v3 (OCA) was used for NGS-based analysis until December 2020, after which the Oncomine Precision Assay was employed.. In addition to these gene panels, the Amoy Master Panel was used in LC-SCRUM-Asia. Of the multiple somatic alterations analyzed in these panels, 20 mutations, which have been reported to be associated with pathogenesis of lung cancer and to have the corresponding therapeutic agents, were reported. These mutations include *RET*, *ALK*, *ROS1*, *NTRK*1–3, and *NRG*1 fusion genes; *FGFR*1–4 gene mutations, amplification, and fusions; *MET* and *ERBB*2 gene mutations and amplification; and *BRAF*, *KRAS*, *NRAS*, *EGFR*, and *PIK3CA* gene mutations [8].

### Statistical analysis

Continuous variables underwent analysis using the Student’s t-test, while dichotomous variables were assessed using the χ2 or Fisher’s exact test, as appropriate.. All *P*-values were two-sided, with *P* < 0.05 considered statistically significant. Pairwise comparisons were performed using Fisher’s exact test with Holm’s adjusted P-values. Logistic regression analysis was used for univariate analysis of factors related to re-genome biopsy; *P* < 0.05 was considered statistically significant. Statistical analyses were performed using EZR software ver 1.29 (Saitama Medical Center, Jichi Medical University, Saitama, Japan) [24].

## Results

### Patient characteristics and sampling methods for genome analysis

**Fig. 1:**
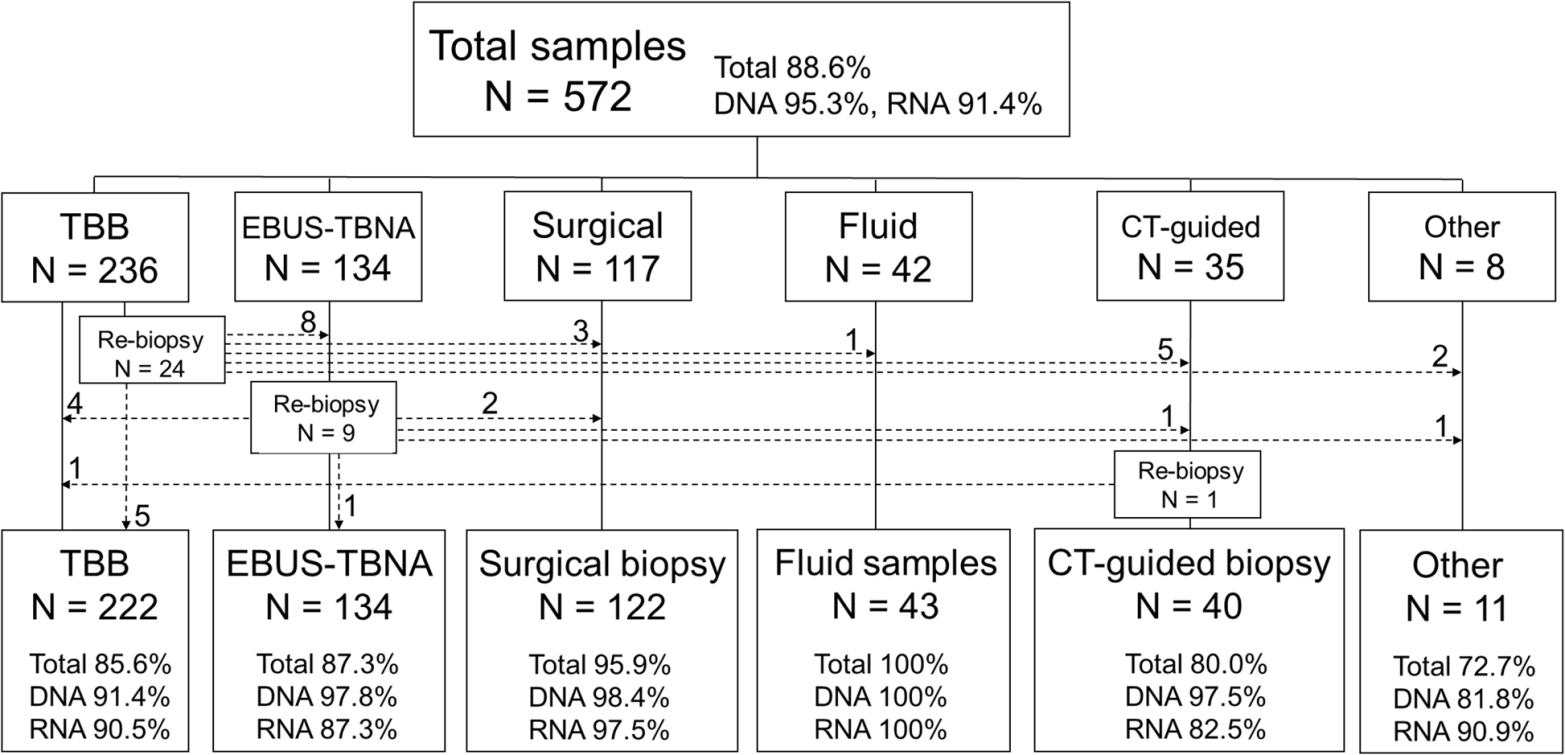
Classification of sampling methods for submitted specimens. A total of 572 samples were biopsied by five methods: transbronchial biopsy (TBB), endobronchial ultrasound with real-time guided transbronchial needle aspiration (EBUS-TBNA), surgical biopsy, fluid sample, computed tomography (CT)-guided biopsy, and others. The success rate of NGS analysis is shown as a percentage, where “Total” is the percentage of success for both DNA-based and RNA-based NGS, “DNA” indicates the success rate of DNA-based NGS, and “RNA” indicates the success rate of RNA-based NGS. The dashed line represents the number of samples for re-genome biopsy

In total, 572 patients from our institution were enrolled in the LC-SCRUM-Asia study. The clinical characteristics of the patients are summarized in Table 1. The median patient age was 68 years (range, 25–93 years). Three hundred and sixty-five patients (63.8%) were male, and the majority (69.2%) had adenocarcinoma, including combined adenocarcinoma with squamous cell carcinoma or small cell carcinoma; 72.7% of the patients had clinical stage IVA or B cancer, whereas the rest were stages IIIA or B (24.5%) and IIA or B (2.8%). The proportion of genome biopsy methods performed for genetic analysis among all cases was 236 for TBB (41.3%), 134 for EBUS-TBNA (23.4%), 117 for surgical biopsy (20.5%), 42 for fluid sample (7.3%), 35 for CT-guided biopsy (6.1%), and 8 for others (1.4%) (Fig. 1). The success rates of the NGS analysis for each sampling method are presented separately for DNA-based NGS and RNA-based NGS, with the combined success percentage shown as “total.”

**Table 1:** Clinical characteristics of patients who underwent genome biopsy with TBB

		Total patients (*n* = 572)	(%)	TBB patients (*n* = 236)	(%)	Re-biopsy in TBB patients (*n* = 24)	(%)
Age-median, [range]	Median	68 [25–93]		68 [25–93]		67 [25–79]	
Sex-no. (%)	Male	365	(63.8)	143	(60.6)	10	(41.6)
	Female	207	(36.2)	93	(39.4)	14	(58.4)
Histology-no. (%)	Ad including	396	(69.2)	160	(67.8)	14	(58.4)
	Ad + SCLC	2		0		0	
	Ad + Sq	2		0		0	
	Sq	99	(17.3)	50	(21.2)	5	(20.8)
	NSCLC	77	(13.5)	26	(11.0)	5	(20.8)
Stage-n. (%)	IVA, B	416	(72.7)	171	(72.5)	21	(87.5)
	IIIA,B,C	140	(24.5)	55	(23.3)	3	(12.5)
	IIA, B	16	(2.8)	10	(4.2)	0	
Tumor location.1	Central			69	(29.2)	14	(58.4)
	Peripheral			167	(70.8)	10	(41.6)
Tumor location.2	Right						
	Upper and Middle			82	(34.7)	10	(41.6)
	Lower			42	(17.8)	6	(25.0)
	Left						
	Upper			67	(28.4)	4	(16.7)
	Lower			45	(19.1)	4	(16.7)
Maximum diameter of tumor.(mm)	Median [range]			39.3 [14.7–102.3]		49.5 [15.6–82.0]	
ROSE	Positive					21	(87.5)

*TBB* transbronchial biopsy, *Ad* adenocarcinoma, *SCLC* small cell lung carcinoma, *Sq* squamous cell carcinoma, *ROSE* rapid-onsite evaluation

### Driver alterations detected in the study

**Fig. 2:**
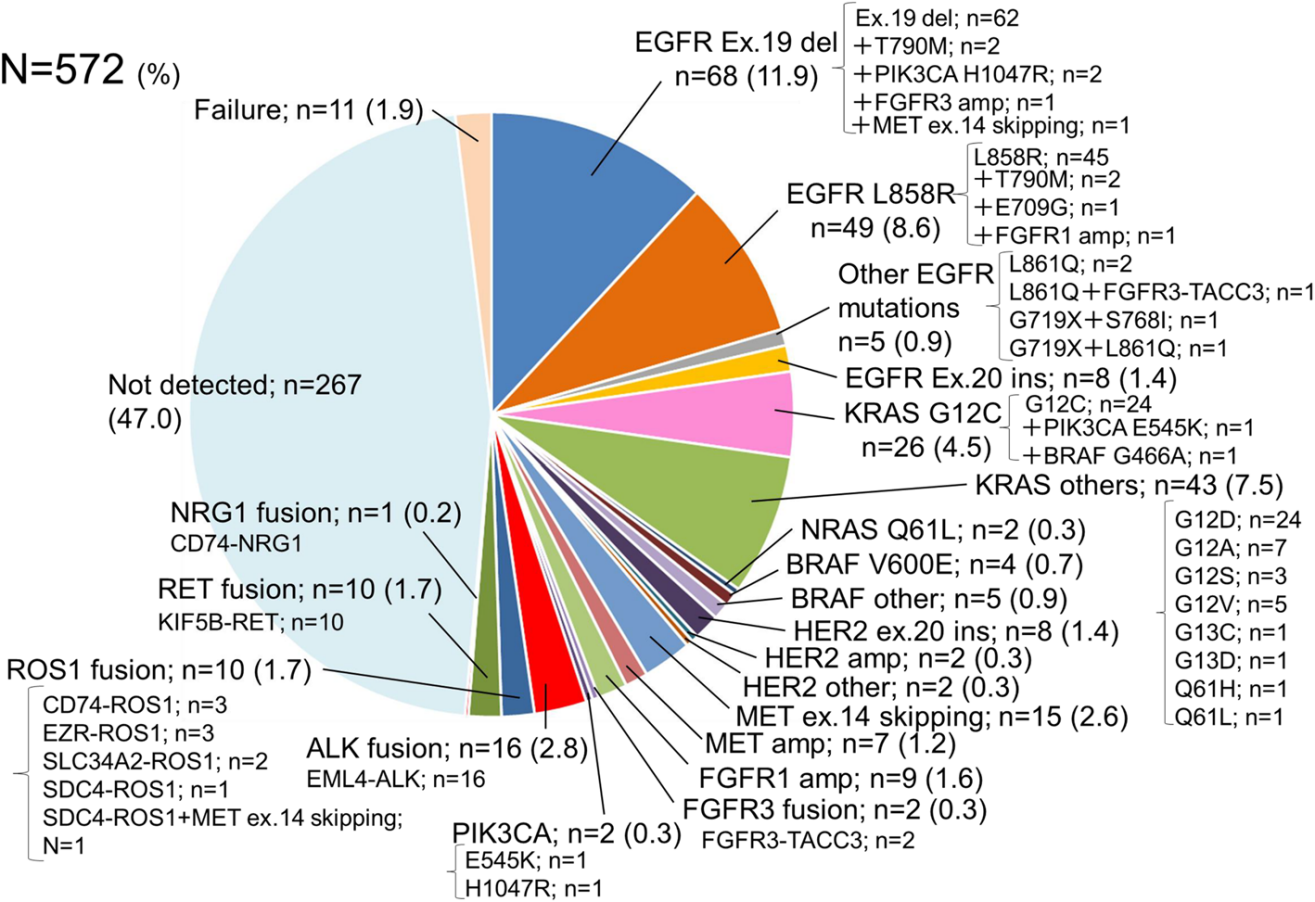
Genetic alterations detected by NGS analysis

The list of driver alterations detected in the 572 specimens is shown in Fig. 2, with 11 cases in which the search for driver alterations could not be performed because both DNA- and RNA-based NGS failed. Some driver alterations were detected in 51.1% of all cases, including those in which either one of the DNA or RNA-based NGS was successful.

### Cases of genome biopsy with TBB and risk of re-genome biopsy

Genome biopsy was performed using TBB in 236 cases, and re-genome biopsy was deemed necessary in 24 cases, based on the assessment of the tumor content of the collected tissue (Fig. 1). The characteristics of each case group are shown in Table 1. Univariate analysis of the risk of re-genome biopsy by clinical factors showed that the risk was significantly increased when the tumor was centrally located (Table 2).

**Table 2:** Analysis of clinical factors for the risk of re-genome biopsy in TBB samples

Variables			Univariate	
		*n* = 236 (%)	OR (95% CI)	*P*
Age, ys	< 75	(77.1)	1	reference
	≥75	(22.9)	0.648 (0.212–1.980)	0.447
Sex	Male	(60.6)	1	reference
	Female	(39.4)	2.360 (0.999–5.560)	0.050
Histology	Adenocartinoma	(67.8)	1	reference
	Others	(32.2)	1.580 (0.667–3.740)	0.298
Tumor location.1	Peripheral	(70.8)	1	reference
	Central	(29.2)	16.60 (5.430–51.00)	< 0.0001
Tumor location.2	Right Upper and Middle/ Lower	(50.9)	1	reference
	Left Upper/ Lower	(49.1)	1.630 (0.692–3.830)	0.264
Maximum diameter of tumor.(mm) [range]	39.5 [14.7–102.3]	(100.0)	1.010 (0.983–1.030)	0.658

*OR* odds ratio, *TBB* transbronchial biopsy

**Fig. 3:**
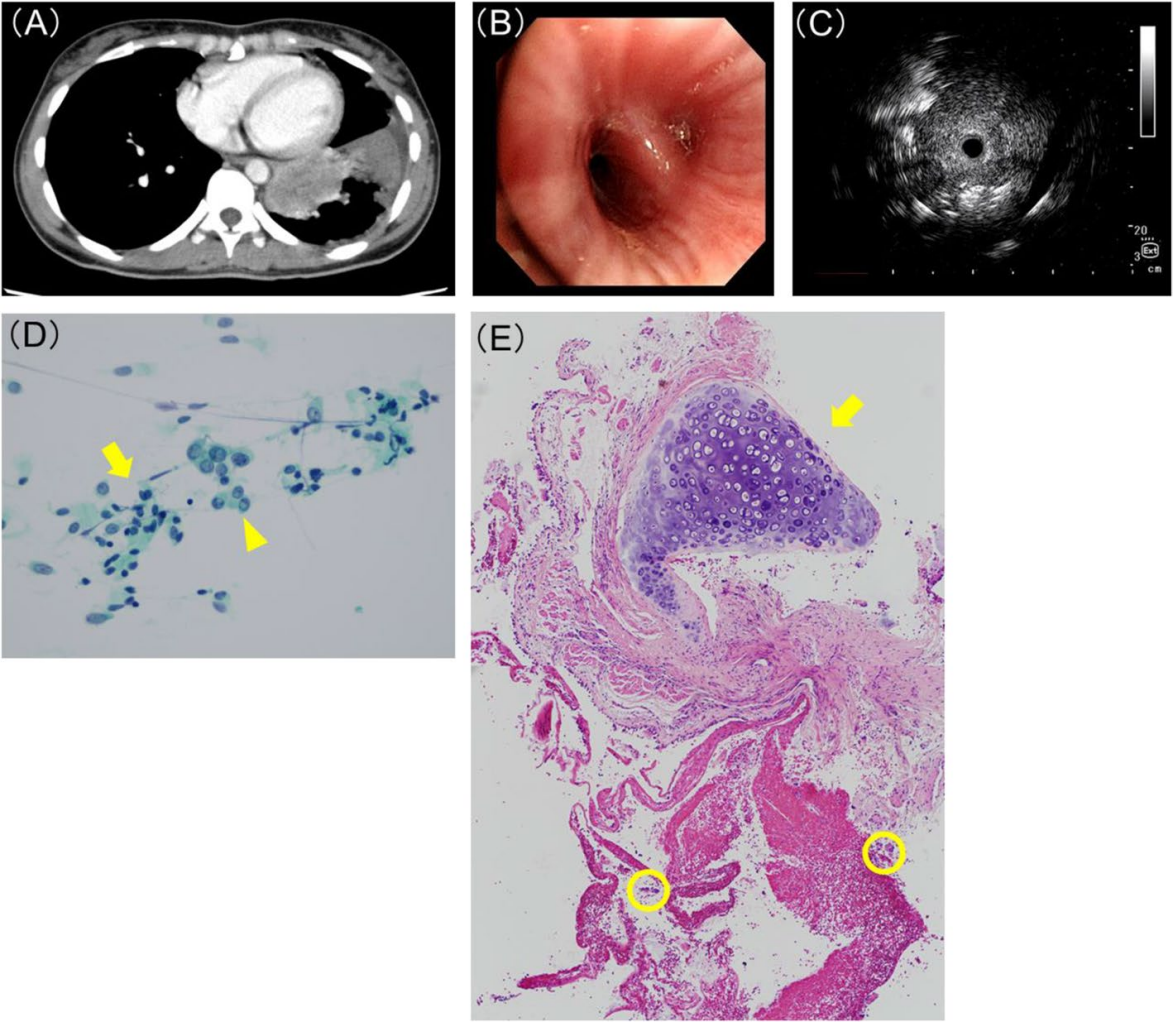
Representative case with a centrally located occupying lesion. A 31-year-old woman with an enhanced chest CT showing a mass shadow in the left lower lobe and a peripheral atelectatic lung formation (**A**). Bronchoscopic luminal finding shows that the lumen of the left lower lobe B9 + 10 is extramurally compressed (**B**). Radial endobronchial ultrasonography (EBUS) probe inserted into the lumen shows an image when the probe is inserted into the tumor (within) (**C**). ROSE finding on TBB forceps smears shows tumor cells with enlarged nuclear dysmorphism (yellow arrow head) and ciliated tracheal epithelial cells (yellow arrow) (**D**). Histopathological analysis of specimen obtained by TBB reveal that tracheal cartilage (yellow arrow) and airway epithelium were mostly present, with only a few clusters of tumor cells (yellow circles)

A representative case is presented herein. A lung mass occupying the left lower lobe towards the center was observed (Fig. 3A), and bronchoscopy revealed narrowing of the left lower B^9+10^ lm (Fig. 3B). Radial EBUS insertion showed an image of an echo probe inserted within the tumor (Fig. 3C). ROSE of the TBB specimen also confirmed malignant cells (Fig. 3D). However, the pathology of the final tissue showed tumor cells in only a small portion, and on the other hand, a significant amount of tracheal epithelium and cartilage was collected (Fig. 3E). Therefore, a re-genome biopsy with EBUS-TBNA was performed, and *ROS1* fusion was successfully detected in this case.

### Cases of genome biopsy with TBB and risk of re-genome biopsy

Genome biopsy using EBUS-TBNA was performed in 134 cases, and their clinical characteristics are shown in Table 3. Of the 134 cases, re-genome biopsy was required in nine cases (6.7%) (Fig. 1). In only one of the nine cases, it was ROSE-negative. Six of 121 mediastinal lymph node lesions (5.0%) required re-genome biopsy, while three of 12 intrapulmonary lesions (25%) required re-genome biopsy. Eight patients who underwent re-genome biopsy with EBUS-TBNA from TBB are not included in Table 3, whose genome analysis was successful in all cases. Of the eight patients with positive ROSE, all had a high percentage of necrosis, tumor content of less than 10%, and were considered unusable for NGS analysis.

**Table 3:** Clinical characteristics of patients who underwent genome biopsy with EBUS-TBNA

		EBUS-TBNA patients (*n* = 134)	(%)	Re-biopsy patients (*n* = 9)	(%)
Age-median, [range]	Median	68 [30–89]		69 [35–73]	
Sex-no. (%)	Male	101	(75.4)	8	(41.6)
	Female	33	(24.6)	1	(58.4)
Histology-no. (%)	Ad	78	(58.2)	5	(58.4)
	Sq	28	(20.9)	2	(20.8)
	NSCLC	28	(20.9)	2	(20.8)
Stage-n. (%)	IVA, B	83	(61.9)	5	(55.6)
	IIIA,B,C	51	(38.1)	4	(44.4)
Tumor location	#2	3	(2.2)	0	
	#4	60	(44.8)	2	(22.2)
	#7	40	(29.9)	3	(33.4)
	#10	4	(3.0)	1	(11.1)
	#11	9	(6.7)	0	
	#12	5	(3.7)	0	
	Upper lobe	4	(3.0)	1	(11.1)
	Lower lobe	8	(6.0)	2	(22.2)
	Other	1	(0.7)	0	
ROSE	Positive			8	(88.9)

*EBUS-TBNA* Endobronchial ultrasound-guided transbronchial needle aspiration, *ROSE* rapid-onsite evaluation

### Number of re-genome biopsies in the first and second half of the analysis period

The period under analysis, January 2019–November 2022, was divided into first and second halves bordering on December 2020, and the proportions of re-genome biopsies were compared for TBB and EBUS-TBNA. In the first half of the year (Jan 2019–Dec 2020) (Table 4), a total of 176 TBBs and EBUS-TBNAs were performed: 126 TBBs and 50 EBUS-TBNAs. In total, 22 re-genome biopsies were performed: 18 TBBs and 4 EBUS-TBNAs. In the second half of the period (Jan 2021–Nov 2022), 194 TBBs and EBUS-TBNAs were performed, 110 were TBBs and 84 were EBUS-TBNAs, with 11 re-genome biopsies for both combined, of which 6 were TBBs and 5 were EBUS-TBNAs. When differences in re-genome biopsy rates were examined, there was a trend toward significantly more re-genome biopsies in the first half of the period and significantly more in the TBB group for both periods together; no differences were found for EBUS-TBNA between the two periods (Table 4).

**Table 4:** Comparison of number of re-genome biopsies in the first and second half of the analysis period

Sampling method	First half year Jan.2019-Dec.2020	Second half year Jan.2021-Nov.2022	Total cases (%)	*P* value
TBB + EBUS-TBNA (n)	176	194	370	
TBB (n)	126	110	236	
EBUS-TBNA (n)	50	84	134	
Rebiopsy in TBB + EBUS-TBNA (%)	22 (12.5)	11 (5.7)	33 (8.9)	*P* = 0.0276*
Rebiopsy in TBB (%)	18 (14.3)	6 (5.5)	24 (10.2)	*P* = 0.0305*
Rebiopsy in EBUS-TBNA (%)	4 (8.0)	5 (6.0)	9 (6.7)	*P* = 0.727

*TBB* transbronchial biopsy, *EBUS-TBNA* Endobronchial ultrasound-guided transbronchial needle aspiration

*indicates statistical significance

## Discussion

Approximately 50% of pretreatment genetic analyses in advanced or locally advanced-stage lung cancer rely on bronchoscopic biopsy [9, 10], in which it is more difficult to obtain sufficient tissue samples in comparison with surgical biopsy. Reducing the risk of re-genome biopsy for genetic analysis is essential for the rapid initiation of treatment and the reduction of patient burden. In this study, we showed that it may be difficult to obtain sufficient tissue samples for genomic analysis by TBB for lesions that centrally occupy. In such cases, the risk of reanalysis can be reduced by actively utilizing needle biopsies such as EBUS-TBNA, even if the lesion is a lung lesion. At the same time, however, it should be noted that pulmonary lesions tend to have a higher risk of re-genome biopsy in EBUS-TBNA compared to lymph node metastatic lesions. Until now, EBUS-TBNA has been performed primarily for metastatic lesions in mediastinal lymph nodes [25, 26], but there have been no reports clearly describing its active utilization for mass lesions in the lung field. Our study is the first report to show that EBUS-TBNA, rather than TBB, is more actively utilized for NGS analysis when a lesion in the lung field is centrally located.

This study showed that ROSE plays a limited role in reducing the risk of re-genome biopsy. Multiple biopsies are required to obtain a sufficient amount of tissue for genome biopsy with TBB, whereas two or more biopsies are required for EBUS-TBNA [10, 27]. It was expected that ROSE could be used to assess the presence or absence of tumor cells without waiting for a histopathological diagnosis [28]. In this study, tumor cells were confirmed using ROSE in 21/24 (87.5%) and 8/9 (88.9%) TBB and EBUS-TBNA re-genome biopsy cases, respectively. Therefore, even if malignant cells are detected by ROSE, it may not be possible to collect a sufficient amount of tissue by genomic biopsy, and the biopsy method may need to be adjusted based on tumor localization.

**Fig. 4:**
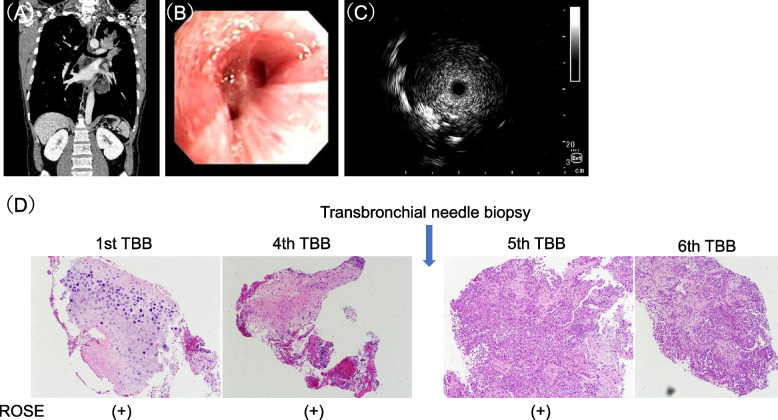
A 45-year-old man with a mass shadow on the left B^1+2^ and bronchoscopic luminal findings showed an image of the left B^1+2^ lm being extramural and compressed. Radial EBUS probe showed an image of a probe inserted into the tumor (within). TBBs were performed, the first to fourth TBBs showed malignant cells in the ROSE ([+] means tumor cell was detected), but the histopathological analysis revealed that tissue specimens did not contain tumor cells; after the fourth biopsy, transbronchial needle biopsy was performed, and the fifth and subsequent biopsies were successful in collecting tissue specimens with a tumor content of more than 80%. Next-generation sequencing analysis detected *EML4-ALK* fusion.Original Article

In the case of pulmonary lesions that are centrally located and externally compress the bronchus, radial EBUS may be inserted into the bronchus responsible for the lesion and produce images similar to those found within the tumor, making the distinction difficult [29, 30]. The results of the present study may reflect the fact that, perhaps because of the influence of mildly exposed lesions in the bronchial lumen, sufficient collection of tumor parenchymal tissue is difficult because of the bronchial wall, even if malignant cells are detected by ROSE in TBB forceps smear specimens. It is hoped that EBUS-TBNA can be utilized in such cases to ensure biopsy of the tumor. NGS analysis was successful in all eight cases in which TBB failed and EBUS-TBNA was used to perform re-genome biopsy. However, there was a trend towards a higher risk of re-genome biopsy for lung lesions in the EBUS-TBNA group. Therefore, EBUS-TBNA is not a satisfactory method. We also reported a case of a centrally located lesion in which neither TBB nor EBUS-TBNA provided adequate tumor samples for NGS analysis. In such cases, other methods, such as the use of cryobiopsy or TBB in combination with TBAC, should be considered. The usefulness of such methods for genomic biopsy of centrally located lung lesions should be investigated (Fig. 4A-D).

EBUS-TBNA for mediastinal lymph node lesions also required re-genome biopsy in six out of 121 cases. In five out of six cases, tumor cells were present on ROSE, but sufficient tissue could not be obtained for histopathological review. In the present study, EBUS-TBNA was mainly performed using 22-gauge needles, but it is expected that the amount of tissue that can be collected will be greater with 19-gauge needles, which may also increase the success rate [31]. There are limited data on the amount of tissue that can be collected compared to EBUS-TBNA, but a greater amount of tissue may be collected when forceps (miniforceps and needle forceps) are used in combination with EBUS-TBNA [32–34]. However, the safety of using these devices in needle biopsy of lung lesions has not been studied and thus they should be used with caution.

If tumor tissue samples cannot be obtained by bronchoscopy, genetic analysis of bronchial lavage fluid containing malignant cells may provide an alternative [35, 36]. If a sufficient sample cannot be obtained by bronchoscopy, a more invasive method, such as a surgical biopsy, may be required. However, in advanced-stage lung cancer, a surgical biopsy is often not possible because of the patient’s general condition and the time required to initiate treatment. Liquid samples are not recommended for genetic analysis because of the high possibility of false-negative results, as the tumor cell content in the specimen cannot be assessed, and there is no consensus on the appropriate amount of specimen to submit. However, if re-genome biopsy is difficult or treatment is urgent, based on the results of ROSE, it is possible to consider storing tracheal and device wash solutions in nucleic acid protection solution or similar. In Japan, the Oncomine Dx® Target Test Multi-CDx System (Thermo Fisher Scientific Inc., Massachusetts, USA) [37] and AMOY Dx® Pan Lung Cancer PCR panel (Amoy Diagnostics Co., Ltd., Xiamen, China) [38], which are gene panels mainly based on tumor tissue samples, have been approved for pre-treatment use in lung cancer in clinical practice, while Lung Cancer Compact Panel™(DNA Chip Research Inc., Tokyo, Japan) using cytology samples has recently been approved. The use of bronchial lavage fluid using ROSE should also be considered for rapid detection of targetable driver alterations.

This study had several limitations. First, being a single-center, retrospective study, the generalizability of the results might be limited due to biases in the TBB and EBUS-TBNA methods and the number of specimens collected. Second, imaging assessment based on the definition of central lesions was not performed in blinded independent central reviews, which does not ensure objectivity in assessing the site of lesion occupation. Third, the NGS analysis in this study was conducted as part of the LC-SCRUM Asia project, and mainly utilized fresh-frozen specimens. Fourth, to confirm the results of this study that EBUS-TBNA is more suitable for NGS analysis than TBB in lesions that centrally occupy, validation in a multicenter prospective study is needed. A common understanding of the definition of centrally location and a uniform method of approach in EBUS-TBNA is needed. Fifth, the results of this study differ slightly from those of clinical practice, as in practice, it is recommended that FFPE specimens be used to assess tumor content before NGS analysis is performed.

## Conclusion

For successful NGS-based gene panel testing, it is necessary to ensure that sufficient quantities of tumor tissue samples are collected, unlike biopsies for diagnostic purposes. It is important to use needle biopsies such as EBUS-TBNA for centrally occupying lesions, even if they are pulmonary lesions, in order to allocate a re-genome biopsy. As the success rate of genome biopsy can be improved with experience, it is expected that cryobiopsy and EBUS-TBNA using mini-forceps, which could not be examined in this study, will further enable the collection of sufficient tissue samples and reduce the risk of re-genome biopsy.

## Data Availability

The data that support the findings of this study are not publicly available due to their containing information that could compromise the privacy of research participants but are available from the corresponding author (Kei Kunimasa, kei.kunimasa@oici.jp) upon reasonable request. Further enquiries can be directed to the corresponding author.

## Abbreviations

NGS: Next-generation sequencing
LC-SCRUM: The lung cancer genomic screening project for individualized medicine
TBB: Transbronchial biopsy
EBUS-TBNA: Endobronchial ultrasound-guided transbronchial needle aspiration
RT-PCR: Multiplex real-time and reverse-transcription-polymerase chain reaction
EGFR: Epidermal growth factor receptor
TKI: Tyrosine kinase inhibitor
CT: Computed tomography
RTOG: Radiation Therapy Oncology Group
ROSE: Rapid on-site evaluation
RET: Rearranged during transfection
ALK: Anaplastic lymphoma kinase
ROS: ROS proto-oncogene
NTRK: Neurotrophic receptor tyrosine kinase
NRG: Neuregulin
FGFR: Fibroblast growth factor receptor
MET: MET proto-oncogene
ERBB2: Erb-b2 receptor tyrosine kinase 2
BRAF: B-Raf proto-oncogene
KRAS: Kirsten rat sarcoma
NRAS: Neuroblastoma rat sarcoma
PIK3CA: Phosphatidylinositol-4,5-bisphosphate 3-kinase catalytic subunit alpha
